# Anti-complement factor H (CFH) autoantibodies could delay pristane-induced lupus nephritis

**DOI:** 10.1007/s12026-023-09396-y

**Published:** 2023-06-16

**Authors:** Lin-Lin Li, Zhong-qiu Luan, Ying Tan, Hui Wang, Xiao-Juan Yu, Zhen Qu, Feng Yu, Min Chen

**Affiliations:** 1 Renal Division, Department of Medicine, Peking University First Hospital, Beijing, China; 2 Renal Division, Henan Provincial People’s Hospital, Zhengzhou, China; 3 Department of Nephrology, First Affiliated Hospital of Heilongjiang, University of Chinese Medicine, Beijing, China; 4 Laboratory of Electron Microscopy, Pathological Centre, Peking University First Hospital, Beijing, China; 5 Department of Nephrology, Peking University International Hospital, Beijing, China

**Keywords:** Lupus nephritis, Pristane-induced lupus, Complement factor H (CFH), Anti-CFH autoantibodies, Protective antibody, Complement activation

## Abstract

**Purpose:**

Anti-complement factor H (CFH) autoantibodies could be detected in lupus and its significance remained to be elucidated. Herein, we aimed to explore the roles of anti-CFH autoantibodies based on pristane-induced lupus mice.

**Methods:**

Twenty-four female Balb/c mice were randomly divided into four groups, with one group injected with pristane (pristane group), one group with pristane and then human CFH (hCFH) (pristane-CFH group) 3 times, and the other two as vertical controls, PBS group and PBS-CFH group. Histopathological analysis was performed six months after pristane administration. Levels of hCFH, anti-CFH autoantibodies and anti-dsDNA antibody were detected. Murine IgG (mIgG) were purified and cross-reactivity, epitopes, subclasses and functional analysis were further evaluated in vitro.

**Results:**

Immunization with hCFH and subsequent development of anti-CFH autoantibodies significantly attenuated nephritis of pristane-induced lupus, including lower levels of urinary protein and serum creatinine, decreased levels of serum anti-dsDNA antibody, greatly ameliorated renal histopathologic damage, decreased IgG, complements (C1q, C3) deposits and lower inflammatory factor (IL-6) expression in glomerulus. Furthermore, the purified mIgG (contained anti-CFH autoantibodies) could recognize both hCFH and murine CFH, and the epitopes were predominantly located in hCFH short consensus repeats (SCRs) 1–4, 7 and 11–14. The IgG subclasses were predominant IgG1. The autoantibodies could enhance the binding between hCFH and C3b, and increase factor I mediated-C3b lysis in vitro.

**Conclusion:**

Our results suggested that anti-CFH autoantibodies could attenuate pristane-induced lupus nephritis by increasing bio-functions of CFH on regulating complement activation and controlling inflammation.

**Supplementary Information:**

The online version contains supplementary material available at 10.1007/s12026-023-09396-y.

## Introduction

Lupus nephritis (LN) is an immune complexes-mediated glomerulonephritis and the most important risk event of morbidity and mortality in systemic lupus erythematosus (SLE) [1]. It was reported that there were nearly 180 autoantibodies in SLE/LN [2]. Autoantibodies against several complement components, including C1q, mannose-binding lectin (MBL), C3b, ficolins-2, ficolins-3 and complement factor H (CFH) have been described in SLE/LN [3–8].

CFH is the most important regulatory protein in the complement system, which is a 155 kDa glycoprotein composed of 20 repetitive domains termed short consensus repeats (SCRs) [9]. CFH can bind to C3b both in the circulation and on the surface by SCRs 1–4, SCR 11–14 and SCR 19–20, accelerate decay of the alternative pathway C3 convertase (C3bBb) and act as a co-factor for the factor I-mediated proteolytic inactivation of C3b [10–12]. CFH recognizes surfaces mainly via glycosaminoglycan (GAG) chains or sialic cluster by SCR7 and SCR19-20, controlling the over-activation on cell surfaces [13–15]. It was reported that anti-CFH autoantibodies could be detected in SLE patients [7]. Our recent published work showed that anti-CFH autoantibodies were positive in 8.3% of LN patients, patients with anti-CFH autoantibodies presented with milder renal damage, and the purified autoantibodies could enhance the C3b binding and CFI cofactor activity of CFH in vitro, which suggested a protective role in the lupus nephritis [16]. The exact roles in the pathogenesis of LN i*n vivo* remained to be elucidated.

Experimental models have provided important insights into the pathogenesis of lupus. Commonly used models involved genetic alterations that increased susceptibility to lupus and induced lupus murine models, including pristane-induced lupus nephritis [17]. Intraperitoneal administration of pristane (2, 6, 10, 14-tetramethylpentadecane), a hydrocarbon oil, was known as the environmentally induced model, which developed several serum autoantibodies (including anti-RNP, anti-dsDNA, anti-ssDNA antibody, etc.) and immune-complex glomerulonephritis [18]. This model has been widely used for exploring the pathogenesis of lupus. In the current study, we explored the potential role of anti-CFH autoantibodies based on pristane-induced lupus nephritis.

## Materials and methods

### Mice

6-8-week-old female Balb/c mice were obtained from the Vital River Laboratory Animal Technology Company (Beijing, China). All mice were housed under specific pathogen-free conditions with controlled humidity and temperature and maintained in a standard 12 h/12 h light/dark cycle with free access to food and water. All studies were approved by the Animal Ethic Committee of Peking University First Hospital (No. 201,815).

### Human CFH (hCFH) and murine CFH (mCFH)

The hCFH was purified from fresh-frozen healthy human plasma as previously described [19], and the concentration of hCFH was detected by ELISA [20].

The mCFH was purified by a modified affinity method using ELISA [3]. Sheep anti-CFH antibody (1:4000) (Abcam, Cambridge, UK) was coated on the plates, then mice plasma (1:50) was added and incubated overnight at 4 ◦C. After washing 3 times, the mCFH was eluted by 0.05 mol/L glycine-HCl (pH 2.7) and neutralized by 0.1 mol/L Tris-HCl (pH 9.0) to pH 7.0, then concentrated and dialyzed against PBS.

The purity of hCFH and mCFH was confirmed by SDS-PAGE and stored at -70 ◦C until use.

### Experimental design

To induce lupus nephritis, mice received a single intraperitoneal (*i.p.*) injection of 0.5 ml of pristane (Sigma, St. Louis, Mo) as previously [18]. Twenty-four female Balb/c mice were randomly divided into following 4 groups (six animals per group, as shown in Table 1): (1) pristane group (positive control): received a single intraperitoneal (*i.p.*) injection of pristane and then received three hypodermic (*i.h.*) injections of a total volume of 100 µl mixtures (50 µl PBS + 50 µl Freund’s complete adjuvant/CFA); (2) pristane-CFH group: received a single i.p. injection of pristane and then received three i.h. injections of a total volume of 100 µl mixtures (50 µl hCFH in PBS (100 µg) + 50 µl CFA); (3) PBS group (pristane-control): received 0.5 ml of PBS and then received three i.h. injections of a total volume of 100 µl mixtures (50 µl PBS + 50 µl Freund’s complete adjuvant/CFA); (4) PBS-CFH group (pristane-CFH control): received 0.5 ml of PBS and then received three i.h. injections of a total volume of 100 µl mixtures (50 µl hCFH in PBS (100 µg) + 50 µl CFA). All the mice were injected 3 times once a week from the 9th to the 11th week of age after pristine administration, and Freund’s incomplete adjuvant (IFA) was used to replace the CFA in the latter two injections. Blood samples were collected before and after immunization weekly until 1 month after pristane injection and then monthly by caudal vein, and urine samples were collected monthly. All the mice were killed 6 months after pristane administration, and kidneys were collected for further experiments.

**Table 1:** Mice groups explored in this study

Group	First (0.5 ml, *i.p.*)	Second (100 µl, *i.h.*)	Third (100 µl, *i.h.*)	Forth (100 µl, *i.h.*)
Pristane	Pristane	PBS + CFA	PBS + IFA	PBS + IFA
Pristane-CFH	Pristane	100 µg hCFH + CFA	100 µg hCFH + IFA	100 µg hCFH + IFA
PBS	PBS	PBS + CFA	PBS + IFA	PBS + IFA
PBS-CFH	PBS	100 µg hCFH + CFA	100 µg hCFH + IFA	100 µg hCFH + IFA

Notes: All mice were divided into four groups, six mice per group. The 100 µg hCFH was in 50 µl PBS, and mice was injected total three times once a week after pristane administration. CFA: Freund’s complete adjuvant; IFA: Freund’s incomplete adjuvant; *i.p*.: intraperitoneal injection; *i.h.*: hypodermic injection; hCFH: human complement factor H

### Evaluations of serum autoantibodies

Serum levels of anti-CFH autoantibodies were assessed by home-made ELISA [21], and anti-dsDNA antibody was evaluated by an ELISA kit (Cusabio Biotech Co. Ltd, Wuhan, China) according to the manufacturer’s instructions.

### Assessments of proteinuria and serum creatinine

Proteinuria was measured in urine collected monthly and serum creatinine concentrations were assessed before pristane administration and at death, respectively. The proteinuria and serum creatinine were tested using UniCel DxC 600 (BECKMAN COULTER, USA) in Clinical Laboratory of Peking University First Hospital.

### Evaluations of histological parameters

Formalin-fixed, paraffin-embedded, periodic acid-Schiff (PAS) reagent sections (4 μm) of the kidneys were assessed for glomerular abnormalities by a professional pathologist.

Glomerular IgG, C1q and C3 were assessed by immunofluorescence on frozen kidney sections. Frozen-sections were stained with goat anti-mouse IgG (Abcam, Cambridge, UK) or rat anti-mouse C1q (Abcam, Cambridge, UK) or rat anti-mouse C3 antibody (Abcam, Cambridge, UK), and then Alexa Fluor 647-conjugated donkey anti-goat IgG or Alexa Fluor 488-conjugated donkey anti-rat IgG (Abcam, Cambridge, UK) was used as secondary antibodies for immunofluorescence microscopy. At least 20 glomerular cross-sections were assessed per animal, and semi-quantitative scoring of IgG, C1q and C3 deposits were analyzed by Image-Pro Plus 6.0 (Maryland, USA).

Renal CFH, IFN-gamma (IFN-γ) and interleukin-6 (IL-6) deposits were stained by immunohistochemistry as previously described [19, 22]. Semi-quantitative scorings of CFH, IFN-γ and IL-6 deposits were analyzed by Image-Pro Plus 6.0 (Maryland, USA).

### Purification of mIgG and its immunologic characteristics

Mice IgG (mIgG) was purified by a protein G affinity column (GE Healthcare, Piscataway, USA) on AKTA-FLPC system and confirmed by SDS-PAGE. The binding between mIgG and hCFH was performed by ELISA, and binding between mIgG and mCFH was performed by Western blot. The IgG epitopes (including hCFH SCRs1-4, 7, 11–14 and 19–20) and subclasses of mIgG were detected by ELISA as previously [21].

### The bio-functions of mIgG in vitro

Functional analysis of the mIgG was further explored by C3b binding assays and the factor I-mediated C3b lysis assays in vitro, as described in the previous study [21].

### Statistical analysis

SPSS software 22.0 (IBM SPSS Inc., USA) was used for calculations. Results were expressed as the mean ± SEM. The ANOVA test corrected by Bonferroni or Mann-Whitney U test was used. *P* < 0.05 was considered statistically significant.

## Results

### The pristane-induced lupus nephritis

**Fig. 1:**
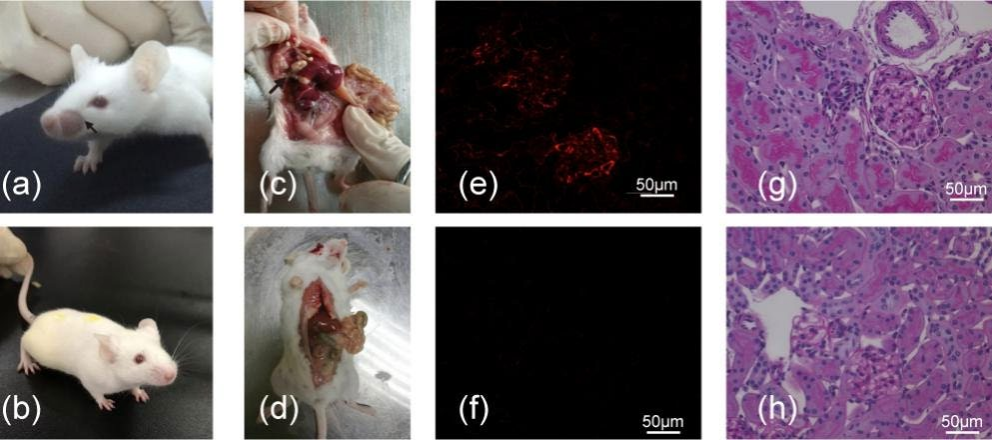
**Pristane-induced lupus mice and control mice**. Appearance of Balb/c mice after intraperitoneal injection of 0.5 ml pristane showing (**a**) depletion of facial hair and (**c**) lymphogranuloma in the peritoneal cavity. Appearance of Balb/c mice after injection of 0.5 ml PBS showing (**b**) normal hair on the face and (**d**) the normal peritoneal cavity without foreign matter(**e**) and (**f**): Representative images of IgG staining in glomerulus in pristane-induced lupus mice (**e**) and control mice (**f**) (original magnifications, x400) (**g**) and (**h**): Representative images of renal damage in pristane-induced lupus mice and control mice (PAS staining, original magnifications, x400): pristane-induced lupus mice presented with severe kidney damage characterized by glomerular mesangial cells proliferation and matrix expansion (**g**); control mice showed normal features of glomeruli, proximal and distal tubules (**h**)

Mice treated with pristane developed a depletion of facial hair, higher levels of serum anti-dsDNA antibodies, lymphogranuloma and renal dysfunction, including significant proteinuria, higher serum creatinine value compared with control mice (Fig. 1, Supplementary Table 1). Obvious IgG deposit by immunofluorescence and severe kidney damage characterized by glomerular mesangial cells proliferation, matrix expansion, and inflammatory cells infiltration by light microscopy were also found in pristane-induced mice but not in the control mice (Fig. 1).

### Measurements of serum and urine parameters in mice

#### Concentrations of hCFH

**Fig. 2:**
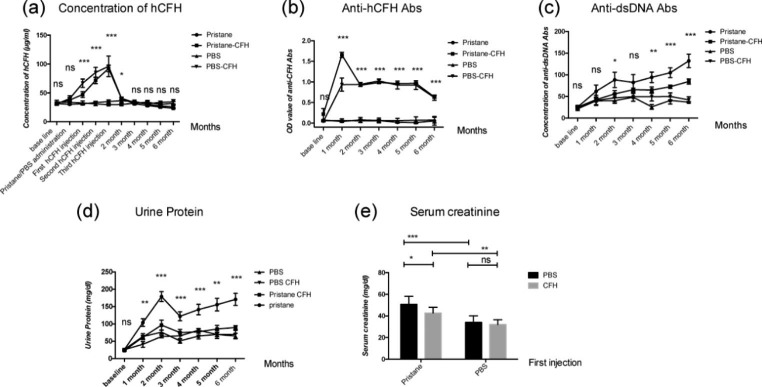
**Assessments of serum and urine parameters in all mice**. (**a**) showed the concentrations of hCFH in all mice. (**b**) showed the OD values of anti-CFH autoantibodies in all mice. (**c**) showed the concentrations of anti-dsDNA antibodies in all mice. (**d**) showed the levels of proteinuria in all mice. (**e**) showed the serum creatinine value of all mice at death 6 month after pristane administration. All mice were divided into four groups, six animals per group. All data were expressed as the mean ± SEM. * <0.05; **<0.01, ***<0.001 and ns = no significance between four groups, respectively

The baseline concentration of hCFH was 32.158 ± 6.044 µg/ml in all mice by ELISA due to the certain reaction between the hCFH and mCFH. The concentrations of serum hCFH increased significantly in the pristane-CFH group and PBS-CFH group after first injection (47.221 ± 4.912 vs. 66.772 ± 7.303, µg/ml, *P* < 0.001), second injection (71.627 ± 5.570 vs. 85.494 ± 8.715, µg/ml, *P* = 0.079) and third injection (92.255 ± 5.504 vs. 96.007 ± 7.797, µg/ml, *P* = 0.759), and turned to the baseline levels (40.029 ± 2.721 vs. 40.412 ± 1.361, µg/ml, *P* = 0.913) one month after the last injection. The levels of hCFH were significantly lower in pristane-CFH group than those in PBS-CFH group at the 5 months and 6 months after pristane administration (25.541 ± 1.230 vs. 33.620 ± 2.155, µg/ml, *P* = 0.005; 23.457 ± 1.040 vs. 34.570 ± 3.741, µg/ml, *P* = 0.009, respectively). The levels of hCFH were stable during the 6 months in the pristane group and PBS group (Fig. 2a, Supplementary Table 1).

#### Levels of serum autoantibodies

Only mice from the pristane-CFH and PBS-CFH group, immunized with hCFH, developed anti-CFH autoantibodies 3 weeks after first hCFH injection. The levels of anti-CFH autoantibodies remained stable five months after pristane administration and then decreased. The levels of anti-CFH autoantibodies in the pristane-CFH group were significantly higher than those in PBS-CFH group at the 1 month after pristane administration (1.656 ± 0.065 vs. 0.926 ± 0.055, *P* < 0.001), and there was no significant difference at other time-points (Fig. 2b, Supplementary Table 1).

Levels of serum anti-dsDNA antibody increased significantly in pristane-induced mice 2 months after pristane administration (Supplementary Table 1). The levels of anti-dsDNA antibody were significantly reduced in mice from pristane-CFH group compared with the pristane group 4 months after pristane administration (*P* = 0.028 at 4 month, *P* = 0.011 at 5 month and *P* = 0.001 at 6 month, respectively) (Fig. 2c, Supplementary Table 1). Anti-dsDNA antibody could not be detected in mice from PBS and PBS-CFH group.

### Assessments of proteinuria and serum creatinine value

Urinalysis revealed that mice from pristane-CFH group had a significantly lower value of proteinuria compared to the pristane group 1 month after pristane administration (Fig. 2d, Supplementary Table 1). There was no significant difference between PBS group and PBS-CFH group (Fig. 2d, Supplementary Table 1).

Mice from pristane-CFH group had a significant decreased serum creatinine value compared with the pristane group at death (42.67 ± 2.201 vs. 50.67 ± 3.062, mg/dl, *P* = 0.034) (Fig. 2e, Supplementary Table 1).

### Evaluations of histological parameters in mice

#### Light microscopy

Mice from the pristane group presented with severe kidney damage characterized by glomerular mesangial cells proliferation, matrix expansion, and inflammatory cells infiltration. No obvious renal damage was found in mice from the PBS/PBS-CFH group.

**Fig. 3:**
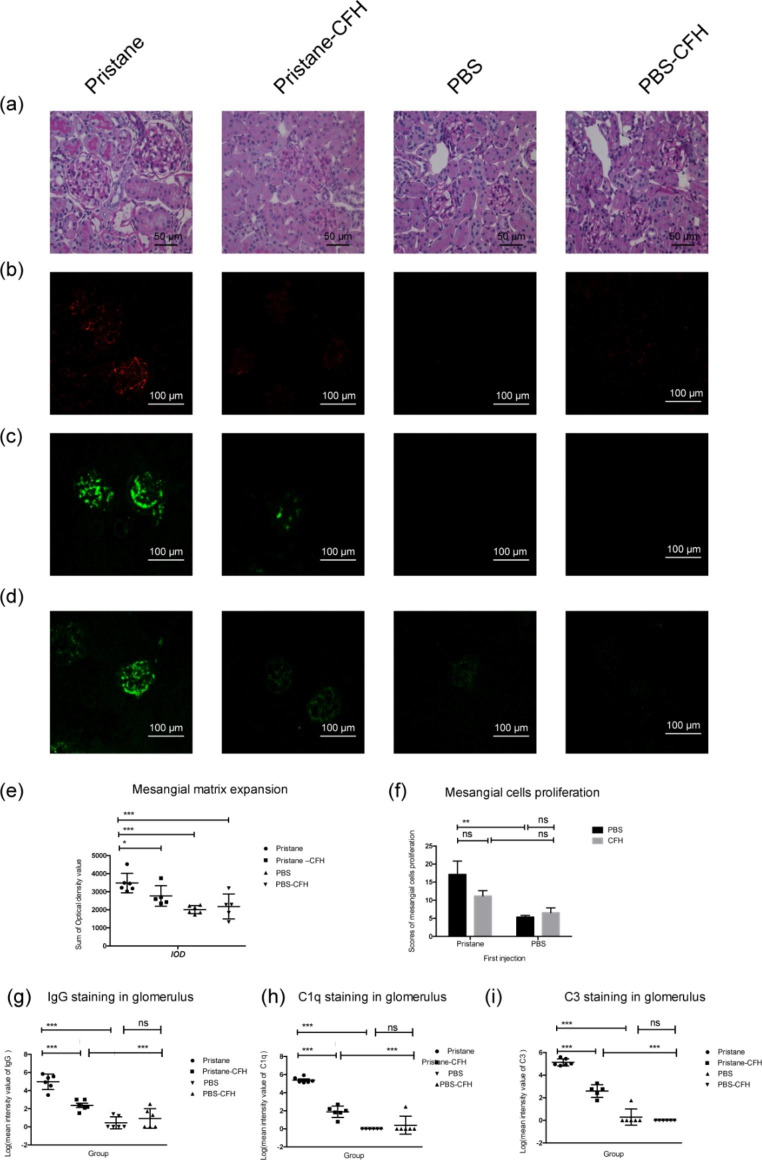
**Renal histological analyses** (**a**): Representative images of renal damage in the four groups (PAS staining, original magnifications, x400): mice from the Pristane group presented with severe kidney damage characterized by glomerular mesangial cell proliferation, matrix expansion and inflammatory cell infiltration; mice from the Pristane-CFH group had milder glomerular mesangial cell proliferation and matrix expansion; mice from PBS/PBS-CFH groups showed normal features of glomeruli, proximal and distal tubules. (**b**), (**c**) and (**d**): Representative images of IgG (**b**), C1q (**c**) and C3 (**d**) staining in glomerular in the four groups (original magnifications, x400). (**e**) and (**f**) showed quantitative analysis for the scores of glomerular mesangial cells proliferation (**e**) and matrix expansion (**f**), respectively. (**g**), (**h**) and (**i**) showed quantitative analysis of immunofluorescence intensities of IgG, C1q and C3 staining in glomerulus, respectively. Six animals per group and all data were expressed as the mean ± SEM

The mice from the pristane-CFH group had significant alleviation in pathological lesions of the glomerulus compared with the pristane group (Fig. 3a). Further quantitative analysis for the scores of glomerular mesangial cells proliferation and matrix expansion supported the results (*P* = 0.038, *P* = 0.173, respectively) (Fig. 3e and f).

### Glomerular depositions of IgG and complements

Mice from the pristane group showed a significant IgG, C1q and C3 deposits compared with the control mice (*P* < 0.001, *P* < 0.001 and *P* < 0.001, respectively) (Fig. 3b, c and d). The significant reduction of IgG, C1q and C3 deposits (*P* < 0.001, *P* < 0.001 and *P* < 0.001, respectively) were observed in mice from the pristane-CFH group compared to the pristane group (Fig. 3g h and 3i).

### Renal CFH, IFN-gamma (IFN-γ) and Interleukin-6 (IL-6) expressions

**Fig. 4:**
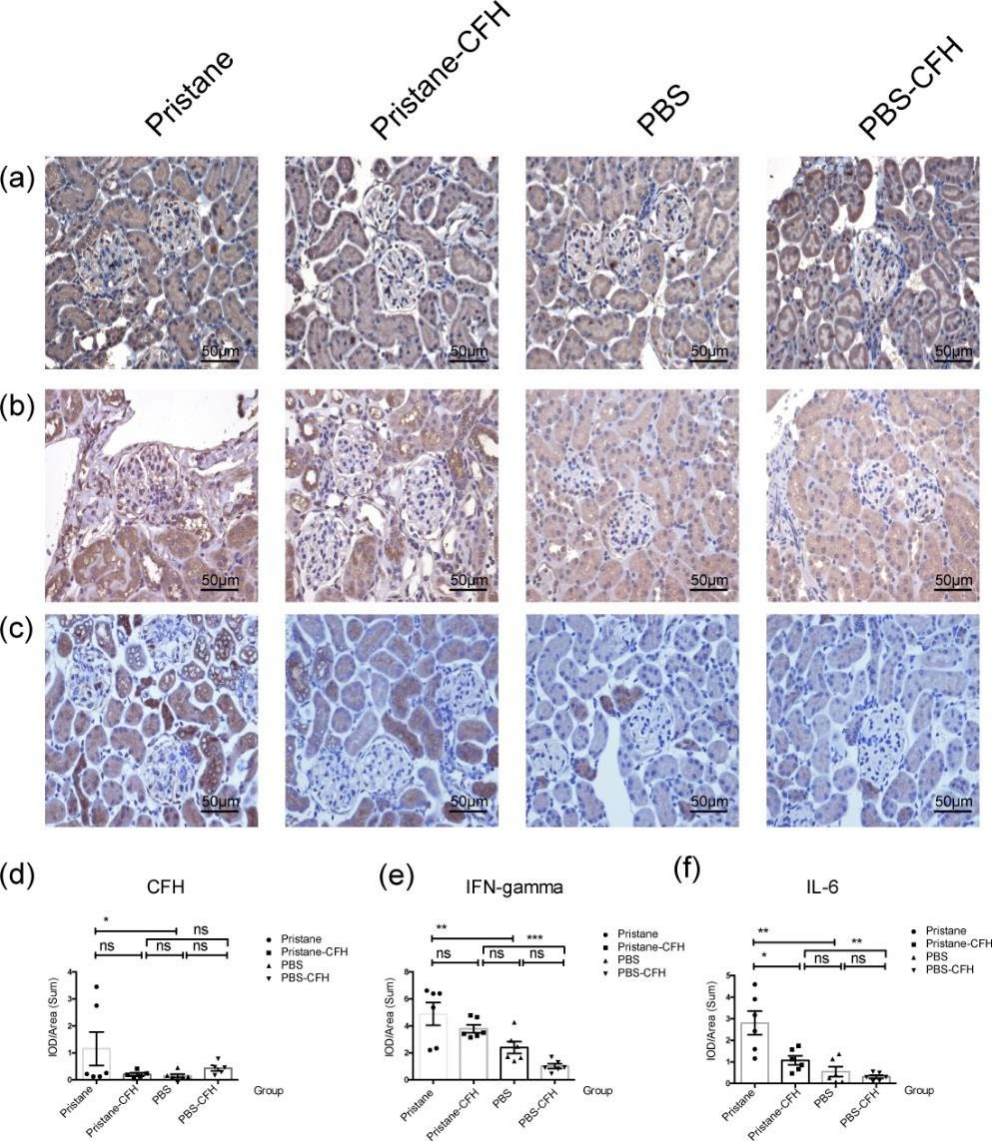
**CFH, IFN-γ and IL-6 deposits in kidney**. (**a**), (**b**) and (**c**): Representative images of renal CFH (**a**), IFN-γ (**b**) and IL-6 (**c**) densities in the four groups, six animals per group. (**d**), (**e**) and (**f**) showed quantitative analysis of intensities of CFH, IFN-γ and IL-6 expression in glomerular, respectively. Six animals per group and all data were expressed as the mean ± SEM, * <0.05; **<0.01, ***<0.001 and ns = no significance, respectively

CFH was mainly expressed in glomerular capillary loop and mesangial areas. The expression of CFH in pristine-induced mice was more dispersive and stronger than those of control mice (*P* = 0.043). The expression of CFH had a lower tendency in the pristane-CFH mice than those in the pristane group (*P* = 0.065) (Fig. 4a and d).

The expression of IFN-γ was significantly higher in mice from the pristane group than the PBS group (*P* = 0.002). There was no significant difference between pristane-CFH group and pristane group (*P* = 0.141) (Fig. 4b and e).

Th*e expression of IL-6 was significantly lower in mice from the pristane-CFH group than those from the pr*istane group (*P* = 0.015) (Fig. 4c and f).

### Correlation analysis between the anti-CFH antibodies and the clinico-pathological parameters in lupus mice

Moreover, the correlation analysis between the anti-CFH antibodies levels and the clinico-histological parameters in pristane-induced lupus mice were further explored displayed in Table 2. We found that the anti-CFH antibodies levels were negatively associated with the proteinuria amount (r=-0.620, *P* = 0.032), the glomerular depositions of IgG, C1q and C3 (r=-0.618, -0.923 and − 0.935 respectively, all *P* < 0.05), and renal IL-6 expressions (r=-0.678, *P* = 0.015).

**Table 2:** Correlation analysis between the anti-CFH antibodies levels and clinico-pathological parameters in lupus mice

	Correlation r	*P* value
Levels of anti-dsDNA antibodies	-0.388	0.212
**Proteinuria amount**	**-0.620**	**0.032**
Serum creatinine value	-0.249	0.435
Scores of glomerular mesangial matrixes expansion	-0.557	0.060
Scores of glomerular mesangial cells proliferation	-0.307	0.331
**Glomerular depositions of IgG**	**-0.618**	**0.032**
**Glomerular depositions of C1q**	**-0.923**	**< 0.001**
**Glomerular depositions of C3**	**-0.935**	**< 0.001**
Renal CFH expressions	-0.398	0.199
Renal IFN-gamma (IFN-γ) expressions	-0.393	0.207
**Renal Interleukin-6 (IL-6) expressions**	**-0.678**	**0.015**

### Immunologic characteristics and functional analysis of anti-CFH autoantibodies in vitro

**Fig. 5:**
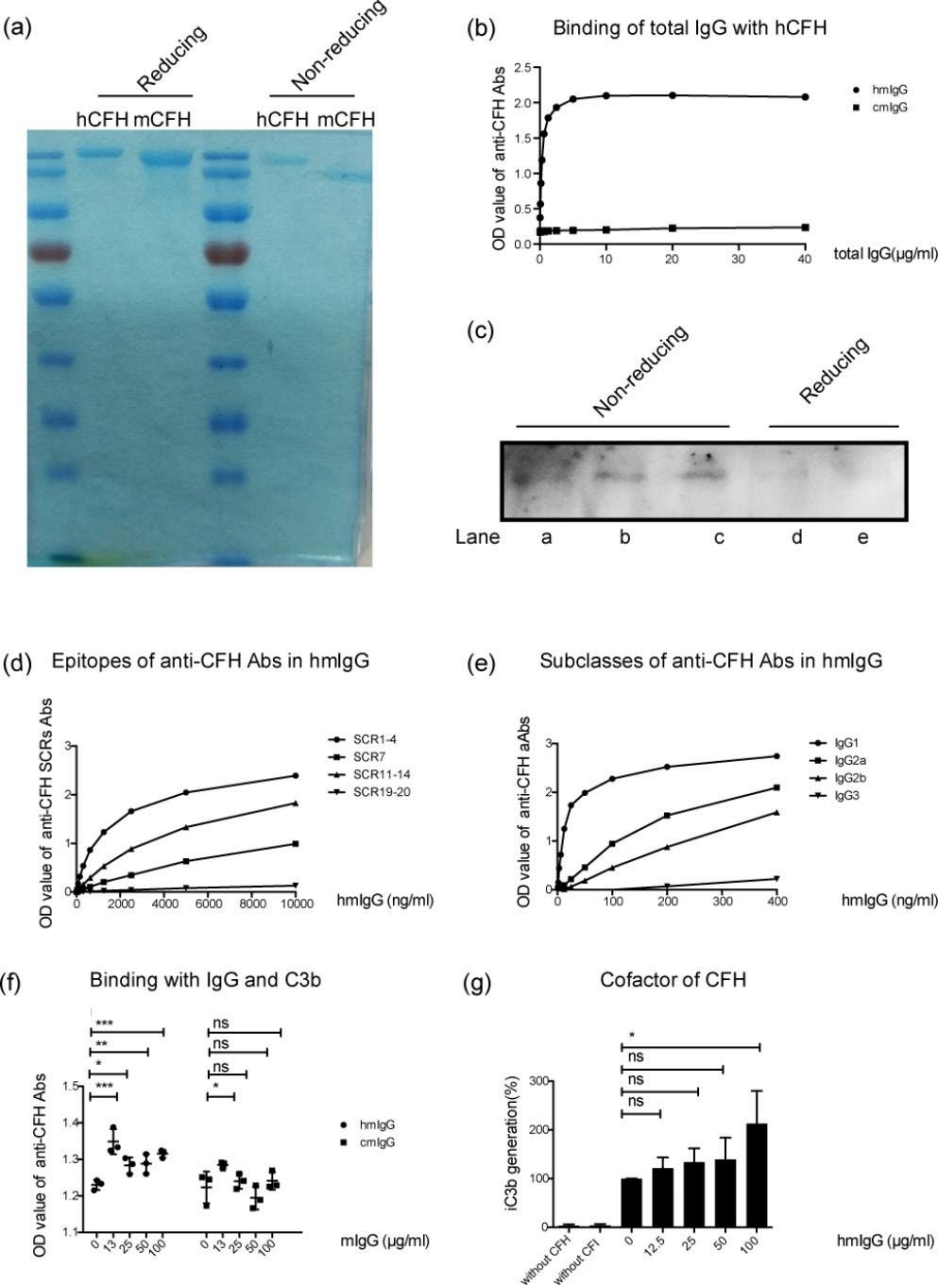
**Presentations of purified hCFH and mCFH, immunological characteristics and functional analysis of the anti-CFH autoantibodies**. (**a**) showed the purity of purified hCFH and mCFH under reducing and non-reducing condition by SDS-PAGE. (**b**) showed the binding between hCFH and mIgG, namely hmIgG and cmIgG, which were purified from mice plasma after immunization with hCFH or PBS. (**c**) showed the binding between hmIgG and mCFH under non-reducing condition. Lanes a, b and c represented mCFH at 0.25, 0.5 and 1.0 µg, respectively, and lanes d and e represented mCFH at 0.25 and 0.5 µg, respectively. (**d**) showed the binding between C3b and hCFH after pre-incubating with hmIgG or cmIgG at different concentrations. Data were expressed as the mean ± SD, and experiments were conducted three individual times. (**e**) showed the CFH cofactor activity of factor I-mediated C3b lysis after pre-incubating with hmIgG or cmIgG at different concentrations. (**f**) showed epitopes of anti-CFH autoantibodies in hmIgG. (**g**) showed the IgG subclasses of anti-CFH autoantibodies in hmIgG. Notes: hCFH: human complement factor H; mCFH: murine complement factor H; hmIgG: total mice IgG purified from mice treated with hCFH and contained anti-CFH autoantibodies; cmIgG: total mice IgG purified mice treated with PBS. Data were expressed as the mean ± SD, * <0.05; **<0.01, ***<0.001 and ns = no significance and experiments were conducted three individual times

The purity of hCFH and mCFH was confirmed in Fig. 5a. Total mIgG after immunizing mice with hCFH or PBS were purified, and we found that the purified mIgG from the PBS-CFH group (hmIgG) could recognize hCFH while the mIgG from the PBS group (cmIgG) couldn’t (Fig. 5b). The hmIgG could also recognize mCFH (Fig. 5c).

The immunologic features, including IgG epitopes and subclasses, of the anti-CFH autoantibodies in the hmIgG were detected. We found that the epitopes were predominantly located in hCFH SCRs1-4, 11–14 and 7, but not SCR19-20 (Fig. 5d). The IgG subclasses of anti-CFH autoantibodies were mainly IgG1, 2a, and 2b, but not IgG3 (Fig. 5e).

HmIgG could enhance the binding between hCFH and C3b (*P* < 0.05) and promote the co-factor activity of CFH on the factor I-mediated C3b lysis in a dose-dependent manner (*P* = 0.032 at 100 µg/ml) (Fig. 5f g).

## Discussion

Autoantibodies against complement factor H (CFH), the key regulator of alternative complement pathway, have been described as being response for atypical hemolytic and uremic syndrome (aHUS) [23], as well as being implicated in the development of C3 glomerulopathies (C3G) [24]. The different binding characteristics, epitopes and *CFHRs* genetic status proposed their heterogeneity between two distinct diseases [25]. Interestingly, anti-CFH autoantibodies could be also detected in patients with systemic lupus nephritis (SLE) and lupus nephritis (LN)[7, 16], and those autoantibodies may be protective in LN in vitro, although their roles in vivo remained to be further elucidated.

Animal models have proved to be suitable tools for the study of SLE/lupus nephritis, including spontaneous lupus model and induced-lupus model. We used a pristane-induced lupus mice model to assess the effects of anti-CFH autoantibodies in vivo. In our study, we successfully observed increasing levels of serum anti-dsDNA antibody and glomerulonephritis, including significant proteinuria, higher serum creatinine value, renal IgG and complement deposits, which were corresponded to the previous studies [18].

Importantly, we found that immunization with hCFH and subsequent development of anti-CFH autoantibodies significantly attenuated glomerulonephritis on pristane-induced lupus nephritis, like decreased levels of proteinuria and lower serum creatinine value, lower levels of serum anti-dsDNA antibody, greatly ameliorated renal histopathologic damage and decreased IgG, complements and some inflammatory factors deposits in glomerulus. Especially, the correlation analysis in pristane-induced lupus mice group further supported the negative association between anti-CFH antibodies and lupus disease activity. Interestingly, those above animals’ results were also consistent with our previous findings in human lupus nephritis cohort [16]. In briefly, the prevalence of anti-CFH autoantibodies in patients with lupus nephritis was 8.3%, and patients with anti-CFH autoantibodies had a significantly lower prevalence of acute kidney injury (0% (0/10) vs. 40.0%(4/10), P = 0.025), lower serum creatinine levels (0.76 (0.40, 1.06) vs. 1.43 (0.46, 11.15), mg/dL, *P* = 0.023), and higher hemoglobin levels (113.8 ± 24.63 vs. 90.0 ± 22.53, g/L, *P* = 0.037) than those who were negative after further stratified analysis, which suggested a protective role in lupus nephritis.

CFH deficiency has been observed to be associated with several glomerular and infectious diseases, including aHUS, C3G and acute infections with *Neisseria meningitides, etc.* [26–28]. The work by Bao et al. also found that CFH deficiency could accelerate the development of lupus nephritis in MRL/*lpr* mice [29]. Importantly, substitution of CFH by means of periodic fresh frozen plasma (FFP) infusion has been suggested as a good therapeutic alternative for HUS patients [30]. The serum half-life of hCFH in mice was influenced by the degree of C3 activation but undetectable by 8 days independent of complement activation [31], which was corresponded to our results and was similar with the 6 days in an aHUS patient [32]. Administration of hCFH to Balb/c mice might restore the complement activation in a short-term (within 1 month hCFH injection) due to the similar functions as mCFH but fail to act in a long term ( 1 month after hCFH injection ) due to its half-life and immune response [31]. Thus, we thought that the subsequent developed anti-CFH autoantibodies might play the long term protective role in the chronic disease process of pristane-induced lupus mice other than the injection of hCFH.

In the current work, we found decreased IgG, C1q, C3 and CFH deposits in glomerular areas in the Pristane-CFH group than disease control, which indicated less complement activation in kidneys. Lupus nephritis was thought to involve glomerular inflammation induced by immune complexes and complement deposition [33], and patients with IgG, C1q and C3c kidney deposition had more severe renal lesions and correlated closely with the clinical disease activity and renal outcomes in lupus nephritis [34]. Less complement activation and inflammation response might result in milder renal pathological lesions in our current study. Importantly, the expressions of IL-6 in renal tissues were decreased in the Pristane-CFH group than disease control. IL-6 played an important role in the progression of lupus nephritis [35, 36]. Several studies reported that blocking IL-6 with anti-IL-6 antibodies or knockout of IL-6 significantly improved survival and delayed nephritis [37–39], whereas mice with IL-6 over-expression developed mesangial proliferative glomerulonephritis [40, 41]. The decreased IL-6 expression might also support the milder renal histopathological features in our current work.

To further clarify the protective roles of anti-CFH autoantibodies, we purified total IgG from mice immunized with hCFH (hmIgG), which was proved to contain anti-CFH autoantibodies, and found that the anti-CFH autoantibodies could recognize both hCFH and mCFH. They modified the bio-functions of CFH in regulating complement activation in vitro, including the binding between CFH and C3b, and factor I-mediated C3b lysis, which might be associated with better control of complement alternative pathway activation.

The immunologic characteristics of anti-CFH autoantibodies, including its epitopes and IgG subclasses, were also analyzed in our study. First, we found that the epitopes were predominantly located in SCRs1-4, then 11–14 and 7, but not SCR19-20. CFH circulates in the blood and controls C3b amplification in the fluid phase usually by its N-terminus, which could bind to C3b, accelerate decay of the alternative pathway C3 convertase (C3bBb) and act as a co-factor for the factor I-mediated proteolytic inactivation of C3b [10–12]. Second, the IgG1 was found to be the main subclass of anti-CFH autoantibodies, then IgG2a and 2b, but no IgG3. Previous work reported that IFN-γ, the prototype Th1 cytokine, promoted the production of IgG2a and IgG3, and played a critical role in the pathogenesis of murine SLE. IL-4, the Th2 cytokine, promoted the switching to IgG1, which was the least pathogenic IgG subclass [42]. The protective role of IgG1 in murine was also observed in other diseases, like cryoglobulinaemia [43]. IgG2a and 2b binds to all types FcγRs while IgG1 only to the inhibitory FcγRIIB and FcγRIII [44]. More importantly, murine IgG1 could inhibit the binding of IgG2a, IgG2b, and IgG3 to C1q in vitro, and suppress IgG2a-mediated complement activation in a hemolytic assay in an antigen-dependent and IgG subclass-specific manner [45]. These might highlight some clues for the protective roles of anti-CFH autoantibodies in our work. It should be noticed that some differences existed in the bio-functions and immunologic features of the anti-CFH autoantibodies between lupus nephritis model and human aHUS and C3G, in which the latter presented with more pathogenic effects [21]. The comparisons of the autoantibodies from varied diseases are needed in the further explorations.

Complement-targeted therapeutics has long been considered a “mission impossible”. However, until early 2023, the approved complement therapeutics now includes the anti-C5 mAbs eculizumab (Soliris, Alexion) and ravulizumab (Ultomiris, Alexion), the C3-inhibitory peptide pegcetacoplan (Empaveli/Aspaveli, Apellis), the C5a receptor 1 antagonist avacopan (Tavneos, Chemocentryx/Vifor), the anti-C1s mAb sutimlimab (Enjaymo, Sanofi), etc., and patients, including lupus nephritis patients, may benefit from those biological agents [46]. Interestingly, our current work suggested that anti-CFH autoantibodies could increase the bio-functions of CFH on regulating complement activation to get better control of complement cascade and substantially alleviate animal’s glomerulonephritis, which may add some information on potential therapeutic impact of complement for lupus nephritis management.

Some limitations did exist in our study. First, we immunized mice with hCFH other than mCFH due to the lack of enough mCFH, although we found a certain cross-reaction between hCFH and mCFH. The further well-defined controlled experiment to eliminate the effects of species specific reactions are needed in the future. Second, we used mice total IgG which proved to contain anti-CFH autoantibodies rather than specific antibodies for further experiments. More studies, including the direct effects of anti-CFH autoantibodies on spontaneous or induced lupus model with CFH deficiency, are needed. Third, we just focused on expressions of IL-6 and IFN-γ by immunohistochemistry in inflamed kidneys, and more in-deep researches at molecular level deserve more attention, e.g. T cells, B cells or macrophage cell infiltration, abnormalities of inflammatory cytokines or chemokines (such as TNF-α, IL-10, IL-1β, IL-17, MCP-1, MIP-1, CXCL13, *ect.*) that recruit T, B, or macrophage in inflamed kidneys and so on. Further renal proteomic and transcript levels of such cytokines or chemokines may provide clear clues for the pathogenesis of LN.

In summary, immunization with CFH and subsequent development of anti-CFH autoantibodies had significantly protective effects on murine lupus nephritis, which might be due to its increased bio-functions of CFH on regulating complement activation and controlling inflammation. More in-deep and rigorous studies at molecular level are needed in further.

### Electronic Supplementary Material

Below is the link to the electronic supplementary material.

Supplementary Material 1
