# Supplementary material for: Anti-complement factor H (CFH) autoantibodies could delay pristane-induced lupus nephritis
Source: Immunol Res. 2023 Jun 16;71(6):849–59. doi: 10.1007/s12026-023-09396-y (PMC10667379; doi:10.1007/s12026-023-09396-y)
Supplement: Supplementary file 1 — Supplementary Material 1 [file 12026_2023_9396_MOESM1_ESM.docx]

**Supplementary Table 1. The levels of serum and urine parameters in all mice explored in this study.**

|  | Pristane | Pristane-CFH | PBS | PBS-CFH | P value | | | | |
| --- | --- | --- | --- | --- | --- | --- | --- | --- | --- |
|  |  |  |  |  | Pristane  /Pristane-CFH | Pristane /PBS | | Pristane-CFH /PBS-CFH | PBS  /PBS-CFH |
| CFH (μg/ml) |  | | | | | | | | |
| Base line | 31.532 ± 3.078 | 32.151 ± 2.135 | 33.928 ± 3.396 | 31.154 ± 3.313 | 0.871 | | 0.553 | 0.804 | 0.492 |
| Pristane injection | 30.139 ± 0.788 | 37.520 ± 4.351 | 33.597 ± 3.847 | 42.014 ± 5.565 | 0.186 | | 0.528 | 0.436 | 0.152 |
| First hCFH injection | 31.222 ± 1.511 | 47.221 ± 4.912 | 32.541 ± 8.774 | 66.772 ± 7.303 | 0.02 | | 0.836 | < 0.001 | < 0.001 |
| Second hCFH injection | 33.175 ± 2.420 | 71.627 ± 5.570 | 30.901 ± 2.993 | 85.494 ± 8.715 | 0.001 | | 0.753 | 0.079 | < 0.001 |
| Third hCFH injection | 35.073 ± 2.574 | 92.255 ± 5.504 | 29.615 ± 1.489 | 96.007 ± 17.797 | < 0.001 | | 0.64 | 0.759 | < 0.001 |
| 2 month | 36.406 ± 2.849 | 40.029 ± 2.721 | 29.991 ± 2.021 | 40.414 ± 1.361 | 0.288 | | 0.068 | 0.913 | < 0.001 |
| 3 month | 35.036 ± 2.559 | 31.222 ± 1.511 | 32.117 ± 2.314 | 33.306 ± 1.609 | 0.203 | | 0.326 | 0.501 | < 0.001 |
| 4 month | 30.485 ± 1.442 | 28.199 ± 0.992 | 33.921 ± 3.123 | 31.244 ± 4.331 | 0.539 | | 0.388 | 0.436 | < 0.001 |
| 5 month | 27.207 ± 1.567 | 25.541 ± 1.230 | 29.956 ± 2.021 | 33.620 ± 2.155 | 0.5 | | 0.271 | 0.005 | < 0.001 |
| 6 month | 26.019 ± 2.471 | 23.457 ± 1.040 | 32.097 ± 2.936 | 34.570 ± 3.741 | 0.49 | | 0.111 | 0.009 | < 0.001 |
| Anti-hCFH Abs |  | | | | | | | | |
| Base line | 0.073 ± 0.005 | 0.220 ± 0.136 | 0.058 ± 0.008 | 0.039 ± 0.003 | 0.155 | | 0.881 | 0.097 | 0.854 |
| 1 month | 0.043 ± 0.011 | 1.656 ± 0.065 | 0.062 ± 0.017 | 0.935 ± 0.080 | < 0.001 | | 0.784 | < 0.0001 | < 0.001 |
| 2 month | 0.076 ± 0.025 | 0.921 ± 0.028 | 0.057 ± 0.015 | 0.939 ± 0.021 | < 0.001 | | 0.563 | 0.583 | < 0.001 |
| 3 month | 0.063 ± 0.023 | 0.987 ± 0.026 | 0.058 ± 0.012 | 1.023 ± 0.021 | < 0.001 | | 0.867 | 0.234 | < 0.001 |
| 4 month | 0.020 ± 0.005 | 0.958 ± 0.011 | 0..058 ± 0.024 | 0.924 ± 0.043 | < 0.001 | | 0.257 | 0.254 | < 0.001 |
| 5 month | 0.014 ± 0.005 | 0.965 ± 0.013 | 0.069 ± 0.034 | 0.922 ± 0.047 | < 0.001 | | 0.172 | 0.302 | < 0.001 |
| 6 month | 0.056 ± 0.043 | 0.634 ± 0.029 | 0.075 ± 0.028 | 0.618 ± 0.029 | < 0.001 | | 0.689 | 0.765 | < 0.001 |
| Anti-dsDNA Abs (mg/dl) |  | | | | | | | | |
| Base line | 25.624 ± 2.521 | 25.224 ± 4.249 | 21.795 ± 4.263 | 21.843 ± 4.127 | 0.941 | | 0.481 | 0.552 | 0.993 |
| 1 month | 61.940 ± 14.538 | 42,791 ± 3.858 | 39.875 ± 8.767 | 40.916 ± 11.658 | 0.199 | | 0.142 | 0.902 | 0.946 |
| 2 month | 88.400 ± 17.692 | 55.857 ± 7.889 | 40.469 ± 6.453 | 46.952 ± 9.553 | 0.054 | | 0.007 | 0.599 | 0.701 |
| 3 month | 82.363 ± 18.252 | 65.816 ± 3.569 | 48.310 ± 9.009 | 49.436 ± 10.464 | 0.321 | | 0.049 | 0.384 | 0.948 |
| 4 month | 94.305 ± 12.148 | 64.704 ± 7.155 | 26.005 ± 5.649 | 49.350 ± 9.906 | 0.028 | | < 0.001 | 0.255 | 0.091 |
| 5 month | 104.917 ± 11.323 | 72.523 ± 3.737 | 40.835 ± 8.307 | 50.145 ± 8.052 | 0.011 | | < 0.001 | 0.081 | 0.452 |
| 6 month | 132.083 ± 15.442 | 84.607 ± 5.863 | 36.884 ± 1.767 | 42.184 ± 7.085 | 0.001 | | < 0.001 | 0.005 | 0.697 |
| Proteinuria (mg/dl) |  | | | | | | | | |
| Base line | 26.150 ± 2.335 | 23.417 ± 2.218 | 26.483 ± 1.096 | 26.000 ± 3.162 | 0.389 | | 0.915 | 0.436 | 0.883 |
| 1 month | 104.067 ± 10.971 | 62.000 ± 5.337 | 63.833 ± 8.552 | 41.280 ± 8.395 | 0.002 | | 0.003 | 0.113 | 0.087 |
| 2 month | 179.050 ± 14.366 | 96.850 ± 14.265 | 76.283 ± 5.429 | 63.760 ± 3.595 | < 0.001 | | < 0.001 | 0.054 | 0.446 |
| 3 month | 122.250 ± 13.031 | 75.050 ± 9.530 | 51.183 ± 6.409 | 65.760 ± 9.168 | 0.003 | | < 0.001 | 0.526 | 0.324 |
| 4 month | 141.583 ± 15.147 | 77.367 ± 9.638 | 65.150 ± 8.223 | 81.980 ± 3.548 | < 0.001 | | < 0.001 | 0.765 | 0.282 |
| 5 month | 155.783 ± 18.217 | 85.467 ± 10.398 | 78.233 ± 12.774 | 69.760 ± 11.643 | 0.002 | | 0.001 | 0.443 | 0.677 |
| 6 month | 170.933 ± 17.567 | 89.850 ± 5.558 | 65.150 ± 7.959 | 69.960 ± 11.626 | < 0.001 | | < 0.001 | 0.254 | 0.779 |
| Serum creatinine value (mg/dl) | | | | | | | | | |
| Base line | 24.67 ± 0.803 | 24.83 ± 0.703 | 25.00 ± 0.775 | 24.80 ± 0.800 | 0.878 | | 0.758 | 0.977 | 0.860 |
| 6 month | 50.67 ± 3.062 | 42.67 ± 2.201 | 34.00 ± 2.503 | 32.00 ± 2.000 | 0.034 | | < 0.001 | 0.009 | 0.591 |

Notes: The mice were divided into four groups, six mice per group. All data were expressed as the mean ± SEM.
